# Management of acute COPD exacerbations in the internal medicine departments in Israel–a national survey

**DOI:** 10.3389/fmed.2023.1174148

**Published:** 2023-08-24

**Authors:** Amir Bar-Shai, Ophir Freund, Tal Ovdat, Michael J. Segel, Robert Klempfner, Avishay Elis

**Affiliations:** ^1^The Institute of Pulmonary Medicine, Tel Aviv Sourasky Medical Center and Sackler Faculty of Medicine, Tel Aviv University, Tel Aviv, Israel; ^2^The Israeli Center for Cardiovascular Research (ICCR) and Sackler Faculty of Medicine, Tel Aviv University, Tel Aviv, Israel; ^3^Institute of Pulmonary Medicine, Sheba Tel-Hashomer Medical Center and Sackler Faculty of Medicine, Tel Aviv University, Tel Aviv, Israel; ^4^Internal Medicine C, Rabin Medical Center and Sackler Faculty of Medicine, Tel Aviv University, Tel Aviv, Israel

**Keywords:** adherence to guidelines, internal medicine, bronchodilators, hospital, exacerbation (symptom flare up), chronic obstructive pulmonary disease

## Abstract

**Background:**

Chronic obstructive pulmonary disease (COPD) is a major cause of morbidity and mortality. Acute exacerbations of COPD (AECOPD) drastically affect the clinical course of the disease. We aimed to evaluate the treatment of AECOPD in the internal medicine departments in Israel, nationwide.

**Methods:**

The COPD Israeli survey (COPDIS) is the first national survey of patients admitted with AECOPD to internal medicine departments between 2017 and 2019. The survey includes prospective (*n* = 344) and retrospective (*n* = 1,166) data from 13 medical centers. We analyzed the pre-hospital, in-hospital, and pre-discharge care. Hospital evaluation, outcomes and discharge recommendations were assessed as well.

**Results:**

The mean (±SD) age was 74 (±8) years, and 54% were males. 74% had comorbidities, and 88% had a diagnosis of COPD in their history. 70% of the patients received systemic steroids and antibiotics during their hospitalization, yet upon discharge, a lower rate of antibiotics prescription (10%) was found. Treatment with most long-acting bronchodilators dramatically dropped during admission, compared with their pre-hospital use. Overall, a long-acting bronchodilator (LABD) was used by 47% before admission, 28% in-hospital, and was prescribed to 54% at discharge. The discharge plan included a referral to pulmonary rehabilitation in only 11% and a smoking cessation recommendation in 43% of active smokers. The in-hospital mortality was 3% and the 1-year mortality rate was 25%. In multivariate analysis, performing a chest X-ray (adjusted OR 0.64, 95% CI 0.46–0.90) and prescribing LABD at discharge (AOR 0.73, 95% CI 0.57–0.95) were independent predictors for lower 1-year mortality.

**Conclusion:**

Our results demonstrate AECOPD characteristics in Israel, and highlight several important gaps in AECOPD healthcare, which must be addressed to improve patient care.

## 1. Introduction

Chronic obstructive pulmonary disease (COPD) is a major cause of morbidity and mortality worldwide with an increased rate despite health care efforts, financial costs and research (1, 2). Comorbidities, such as heart failure and diabetes mellitus, are frequent among COPD patients and significantly impact their quality of life, exacerbation frequency and survival (3, 4). The most relevant events affecting COPD mortality are acute exacerbations (AECOPD), as their frequency and severity are significant modifiers for management and outcomes (3, 5, 6). Furthermore, exacerbations are the primary cause of hospital admissions and account for 40–75% of COPD’s total health care costs (7, 8).

Although guidelines and standard of care recommendations have been published (3, 5, 9), there are still gaps in the treatment of AECOPD in internal medicine departments, where most of these patients are hospitalized (10–12). We hypothesized that similar gaps exist in the care of AECOPD before and during their hospitalization at the internal medicine departments in Israel. There are no domestic accepted guidelines for the care of AECOPD patients in Israel. The Global Initiative for Chronic Obstructive Lung Disease (GOLD) guidelines are the reference for the standard of care in most hospitals and respiratory clinics in Israel. Our aim was to evaluate the pre-admission, in-hospital, and pre-discharge management of patients with AECOPD on a national scale, with reference to the GOLD guidelines, in order to raise awareness and possibly lead to a change in health policy.

For this purpose, we conducted the Chronic Obstructive Pulmonary Disease Israeli Survey (COPDIS), a national multicenter survey aimed to evaluate the routine care of patients with AECOPD hospitalized at internal medicine departments in Israel. In this manuscript, we describe its results and comparison with the recommended care by the main guidelines.

## 2. Methods

### 2.1. Study design and participates

COPDIS is a survey of patients with AECOPD, who were admitted to 40 internal medicine departments in 13 medical centers across Israel between 2017 and 2019. The current survey is based on two cohorts, one retrospective (*n* = 1,166) and one prospective (*n* = 344). The study was approved by each institutional ethical committee.

We included both cohorts to better represent AECOPD hospitalizations on a national scale. The inclusion criteria were: (1) adults >18 years old, (2) known COPD based on medical history or spirometry tests, (3) admission to internal medicine department, and (4) AECOPD as the main diagnosis given to the patient at discharge with at least one relevant symptom including increased dyspnea, sputum, or cough. Patients without a prior diagnosis of COPD were included only if there was no other condition that explained their symptoms and they were treated as AECOPD during their hospitalization. The in-hospital management was at the discretion of each medical center. In most cases, AECOPD led to hospitalization due to respiratory failure with hypoxemia and/or hypercapnia. Among the other less common indications for hospitalization were concurrent severe pneumonia, high-risk comorbidities, or social reasons.

The retrospective cohort data was extracted from hospitals’ electronic records, followed by revision of a senior physician (respiratory specialist). It included patients’ baseline characteristics (age and comorbidities), chronic treatment prior to admission (such as inhalers or steroids), in-hospital characteristics and management (imaging, initial vital signs and laboratory tests, spirometry, complications, and treatment), and hospital outcomes (hospital stay duration, in-hospital mortality and one-year mortality).

For the prospective data cohort, patients were enrolled during their admission to the internal medicine department for AECOPD after providing written informed consent. Data was extracted using an electronic case report form (CRF) designed by the COPDIS Steering Committee and included similar data as the retrospective cohort with the additional following information: prior COPD history and characteristics (such as a prior exacerbation, symptom severity and pulmonary follow-up), respiratory exposures (environmental and occupational), and the discharge plan (including recommendations and prescriptions at discharge).

### 2.2. Study variables and outcomes

The main outcome was the inpatient care of AECOPD, including short-acting bronchodilators, antibiotics, systemic steroids, and the performance of a chest X-ray. The secondary outcomes were: (1) other COPD-related treatments before admission (chronic treatment), during hospitalization and at discharge, (2) in hospital mortality and hospital stay duration, (3) One-year mortality (assessed using the National Population Registry of the Israel Ministry of the Interior), and (4) the pre-hospital COPD-related management (spirometry, pulmonologist visits, and previous exacerbations). An additional analysis was performed to highlight variables associated with 1-year mortality.

### 2.3. Statistical methods

Patient characteristics are presented as *n* (%) for categorical variables, and as mean (standard deviation, SD) or median (inter-quartile range, IQR) for normally and non-normally distributed continuous variables, respectively. Normality was evaluated using the Kolmogorov–Smirnov test. We calculated the Charlson Comorbidity Index for each patient, which is used to estimate the 10-year survival rate. Variables with >20% of missing data were omitted. Chi-square tests were performed to compare categorical variables. *T*-tests were performed to compare continuous variables. Independent associations between all included patients’ variables and 1-year mortality were analyzed by a multivariate regression model which included age, sex, and the significant relevant variables from a univariate analysis. *p* < 0.05 was required for statistical significance. Data analysis was performed using the IBM SPSS Statistics version 22.

## 3. Results

The baseline characteristics of the entire cohort are presented in Table 1. The overall mean (SD) age was 74 (8) years, 46% were females, and 14% had a concurrent diagnosis of asthma.

**Table 1:** Baseline characteristics of the COPDIS cohort^a^.

Variable	Total *n* = 1,510 (%)
Age, yrs	74 ± 8
Male sex (%)	822 (54)
Comorbidities, *n* (%):
Charlson comorbidity index	5.5 ± 2
Hypertension	770 (51)
Dyslipidemia	579 (38)
Obese (BMI > 30)^b^	428 (32)
Diabetes mellitus	461 (30.5)
Heart failure	404 (27)
Ischemic heart disease	404 (27)
Cancer	299 (20)
Chronic kidney disease	272 (18)
Asthma	208 (14)
Prior stroke	198 (13)
Depression	128 (8.5)
Pulmonary hypertension	77 (5)
Obstructive sleep apnea	49 (3)

^a^Percentages are calculated from the total available records.

^b^Data is missing for 11% of the cohort.

Environmental exposures and COPD-related characteristics, available only for the prospective cohort, are presented in Table 2. Ninety-three percent of the patients were past or current smokers with a median of 50 (IQR 40–80) pack years; 45% were active smokers. Overall, 88% had been diagnosed with COPD prior to the index admission, 78% visited a pulmonologist and 74% had performed spirometry test at least once. Prior COPD exacerbations occurred in 78% of the patients, of whom it led to hospitalization in 82% and mechanical ventilation in 14%. The Modified Medical Research Council (mMRC) score was ≥2 (considered a high symptom burden and a significant disability imposed by breathlessness (3)) among 69% of the cohort. Only 10% performed exercise at least once a week.

**Table 2:** Environmental exposures and COPD characteristics^a^.

Variable	Total *n* = 344 (%)
Exposure characteristics
Smoking	
Never	24 (7)
Past	164 (48)
Current	156 (45)
Pack years	50 (40–80)
Past or current passive smoking	111 (32)
Dust exposure	69 (20)
Asbestos exposure	16 (5)
Metals or chemicals exposure	64 (18.5)
Living next to a main road	171 (50)
COPD characteristics
Known COPD	303 (88)
Prior pulmonologist visit	240 (78)
Prior spirometry	234 (73.5)
Prior COPD exacerbation	243 (78)
Prior COPD hospitalization	198 (64)
Prior mechanical ventilation	34 (11)
mMRC dyspnea scale	
0	56 (15.5)
1	43 (12)
2	55 (15)
3	64 (18)
4	128 (36)
Any Weekly physical activity	34 (10)
Exercise (sessions per week)	3 (2–7)
Activity duration (minutes per session)	40 (30–60)
Duration of symptoms prior to current admission (days)	4 (2–6.3)

COPD, chronic obstructive pulmonary disease; mMRC, Modified Medical Research Council. ^a^Percentages are calculated from the total available records. Data are based on the prospective case report forms and are presented as median (IQR) or n (%).

### 3.1. In hospital clinical characteristics

The in hospital clinical characteristics of the entire cohort are presented in Table 3. Upon admission, patients’ median (IQR) room air saturation was 93% (88–96%). Chest X ray was done in 85% of the cases and chest computed tomography (CT) in 12% (7% chest CT angiography). Spirometry was performed in 81 patients (5%) before discharge. The initial median (IQR) serum eosinophil count was 0.06 K/ul (0.01–0.34). Complications during the hospitalization included lobar pneumonia (13%) and congestive heart failure exacerbation (12%). Less common complications included acute kidney injury (5%), severe sepsis (1%) and deep vein thrombosis (0.5%).

**Table 3:** Admission characteristics and outcomes.

Variable	Total *n* = 1,510 (%)
Initial measurements
O_2_ Saturation (%)	93 (88–96)
Systolic blood pressure (mmHg)	136 (120–155)
Heart rate (bpm)	90 (78–105)
Body temperature (Celsius)	36.8 (36.6–37.2)
In-hospital procedures
Chest X Ray	1,289 (85.4)
Standard Chest CT	77 (5.1)
Chest CT-angiography	100 (6.6)
Spirometry	81 (5.4)
Echocardiography	234 (15.5)
Ventilation-perfusion scintigraphy	7 (0.5)
Initial laboratory results
Creatinine (mg/dL)	0.9 (0.7–1.2)
Hemoglobin (g/dL)	12.7 (11.1–14.1)
White blood cells (K/ul)	10.4 (7.6–19.4)
Neutrophils (%)	78.5 (69.4–86.8)
Eosinophils (%)	0.4 (0.1–1.4)
Eosinophils (K/ul)	0.06 (0.01–0.34)
CRP (mg/L)	24.6 (8.5–86)
In-hospital complications
Lobar pneumonia	198 (13.1)
Congestive heart failure	182 (12.1)
Symptomatic arrhythmia	78 (5.2)
Acute renal failure	69 (4.6)
Severe sepsis	13 (0.9)
Deep vein thrombosis	6 (0.4)
In-hospital outcomes
Hospital duration (days)	2 (1–5)
Death	45 (3)

### 3.2. Chronic obstructive pulmonary disease-related medication trends

**Figure 1:**
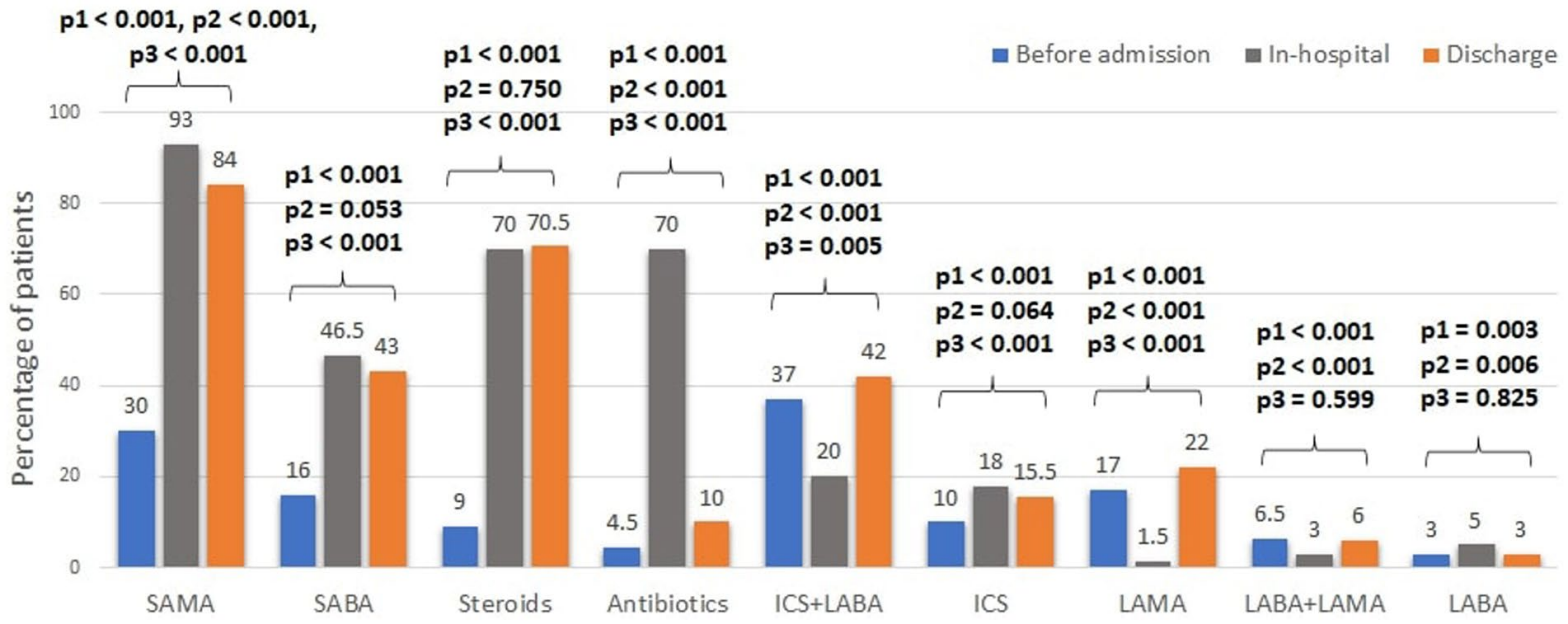
Trends in treatments of acute COPD exacerbations. SABA, short-acting beta agonist; LABA, long-acting beta agonist; SAMA, short-acting muscarinic antagonists; LAMA, long-acting muscarinic antagonists; ICS, inhaled corticosteroids. Steroids refers to any systemic steroids given. * Comparisons between the treatment rates were performed using the Chi-square test and in the following order: p1- between pre-admission and in-hospital; p2- between the in-hospital and pre-discharge; p3- between pre-admission and pre-discharge.

**Figure 2:**
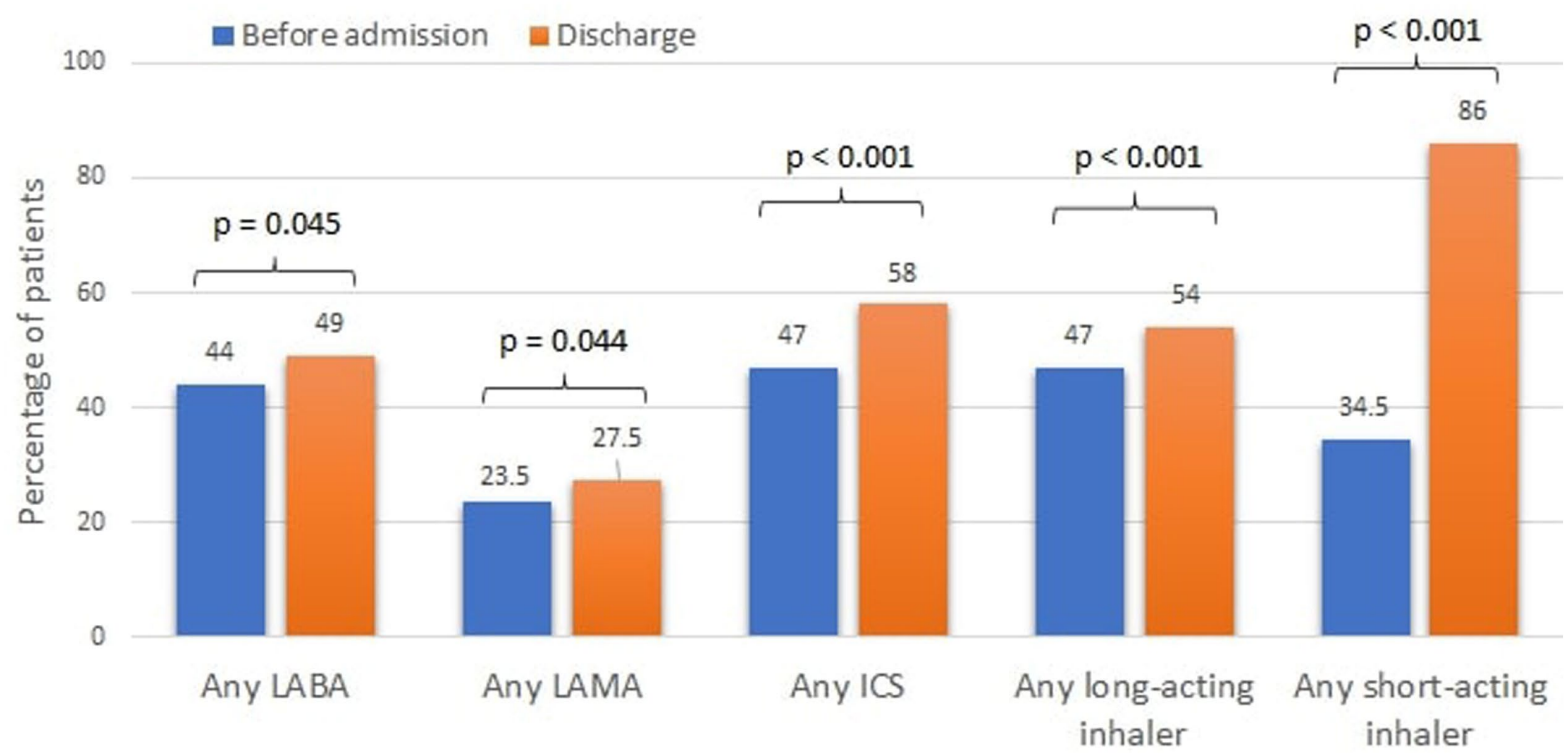
Cumulative inhaler treatment of COPD prior to admission and at discharge. LABA, long-acting beta agonist; LAMA, long-acting muscarinic antagonists; ICS, inhaled corticosteroids.

Inhaled respiratory treatments before, during hospitalization and at discharge are presented in Figures 1, 2. Short-acting muscarinic antagonists (SAMA) were the most frequently used inhaled therapy during admission (93%), while long-acting muscarinic antagonists (LAMA) were the least (1.5%). The treatment rate of most long-acting bronchodilators (LABD) dropped during hospitalization and slightly increased at discharge, including, LAMA (1.5% during hospitalization vs. 17% prior to admission and 22% recommended at discharge), combined long-acting beta-agonist (LABA) with LAMA (3% vs. 6.5 and 6%), and combined inhaled corticosteroids (ICS) with LABA (20% vs. 37 and 42%). Antibiotics and systemic steroids (intravenous or oral) were each given to 70% of the patients during admission.

At discharge, only 54% of the patients were prescribed a long-acting inhaler (LABA, LAMA, LABA+LAMA, ICS + LABA) and only 58% an ICS containing regimen (Figure 2).

### 3.3. Pre-discharge characteristics and outcomes

Based on the prospective data, upon discharge, 37.1% of the patients still required oxygen support and 95% were discharged to their home (Table 4). Only 16.6% were referred to spirometry testing and 69.4% to pulmonologist follow-up. Among active smokers, only 43% had a recommendation for smoking cessation at discharge and 13.5% were referred to a smoking cessation program.

**Table 4:** Discharge characteristics and plan^a^.

Variable	Total *n* = 344 (%)
Respiratory status at discharge:	
No respiratory support	197 (62.9)
Oxygen therapy	85 (27.2)
Intermittent BiPAP	30 (9.6)
Mechanical ventilation	1 (0.3)
Discharge setting:	
Home	304 (95)
Nursing home	9 (2.8)
Recovery unit	6 (1.9)
Recommendations at discharge
Referral to rehabilitation program	37 (10.8)
Referral to smoking cessation program	21 (6.1)
Recommendation for smoking cessation	67 (19.5)
Recommendation for A1AT testing	2 (0.6)
Recommendation for spirometry	57 (16.6)
Recommendation for pulmonologist follow-up	238 (69.4)

BiPAP, bilevel positive airway pressure; A1AT, alpha-1 antitrypsin. ^a^Percentages are calculated from the total available records. Data are based on the prospective case report forms and are presented as *n* (%).

Patient outcomes are provided in Table 3. The median (IQR) hospital stay was 2 (1–5) days. In-hospital mortality was 3%. The one-year mortality rate was 24.9%. Older age (adjusted OR 1.06, 95% CI 1.04–1.07, *p* < 0.01), known heart failure (AOR 1.84, 1.35–2.51, *p* < 0.01), chronic kidney disease (AOR 1.59, 95% CI 1.15–2.22, *p* < 0.01), and prior intubation (adjusted OR 1.84, 95% CI 1.29–2.62, *p* < 0.01) were independent predictors for higher 1-year mortality (Table 5). On the contrary, performing chest X-ray during hospitalization (AOR 0.64, 95% CI 0.46–0.90, *p* = 0.01) and prescribing LABD at hospital discharge (AOR 0.73, 95% CI 0.57–0.95, *p* = 0.02) were independent predictors for lower 1-year mortality. Of note, prescribing short-acting bronchodilators was associated with higher mortality in the univariate analysis, although it was not significant after adjustment in the multivariate model.

**Table 5:** Univariate and multivariate analyzes of predictors for 1-year mortality.

Variables	Univariate		Multivariate	
	OR (95% CI)	*p*	AOR (95% CI)	*p*
Older age	1.06 (1.0–1.1)	<0.01	**1.06 (1.04–1.07)**	**<0.01**
Female sex	1.12 (0.9–1.4)	0.35	0.99 (0.76–1.30)	0.97
Hypertension	1.54 (1.2–2.0)	<0.01	0.85 (0.63–1.14)	0.26
Diabetes	1.35 (1.1–1.7)	0.02	1.18 (0.88–1.60)	0.27
Ischemic heart disease	1.41 (1.1–1.8)	0.01	0.83 (0.61–1.13)	0.24
Heart failure	2.75 (2.1–3.5)	<0.01	**1.84 (1.35–2.51)**	**<0.01**
Chronic kidney disease	2.80 (2.1–3.7)	<0.01	**1.59 (1.15–2.22)**	**<0.01**
Depression	1.62 (1.1–2.4)	0.02	1.30 (0.85–2.01)	0.23
Prior intubation	2.25 (1.6–3.1)	<0.01	**1.84 (1.29–2.62)**	**<0.01**
In-hospital chest X-ray	0.62 (0.5–0.9)	<0.01	**0.64 (0.46–0.90)**	**0.01**
SABD at discharge	1.51 (1.0–2.2)	0.01	1.29 (0.87–1.91)	0.21
LABD at discharge	0.73 (0.6–0.9)	0.03	**0.73 (0.57–0.95)**	**0.02**
ICS at discharge	0.82 (0.6–1.0)	0.10		

AOR, adjusted odds ratio; SABD, short acting bronchodilator; LABD, long-acting bronchodilator; ICS, inhaled corticosteroid.

Bold values signify statistical significant value in multivariate analysis.

## 4. Discussion

We describe the results of the first national Israeli survey of patients who were hospitalized with an AECOPD in internal medicine departments. Our survey provided the following insights: (1) The in-hospital care of most patients complied with the basic elements of the recommended AECOPD management, including chest X ray (85%), antibiotic use (70%), prescription of short acting bronchodilators (98%) and steroid administration (70%). (2) Contrary to the guidelines (3, 13), prescription rates of most LABDs dropped during hospitalization and increased upon discharge, but only to 54% of the patients. (3) Only a minority of the patients were referred for pulmonary rehabilitation (11%), smoking cessation programs (13.5% of active smokers) or spirometry (17%). (4) An overall short hospital duration (median 2, IQR 1–5) with 3% in-hospital mortality and 25% 1-year mortality. (5) In-hospital chest X-ray and prescribing LABD upon discharge were associated with lower 1-year mortality.

The baseline characteristics of our cohort, including age and rates of comorbidities, are in line with previously reported cohorts (14, 15). Among our prospective cohort, 88% had a prior diagnosis of COPD, also similar to published literature (16). There was a high percentage of active smokers (45%) in our prospective cohort compared to previously described (17). Despite most patients had already visited a pulmonologist (78%) and performed spirometry (73.5%), only 47% were on LABD before their admission.

The different aspects of inpatient care among our study population showed a variable adherence to guidelines recommendations. Most guidelines recommend the use of short-acting bronchodilators, systemic steroids, and antibiotics for hospitalized patients with AECOPD (3, 13). In our study, almost all patients were treated with SAMA (93%). However, only 46.5% received SABA, which is lower than most previous studies of inpatient AECOPD management (16–18). The low rate of SABA administration might be a result of reluctance to prescribe SABA among patients with a history of heart failure or ischemic heart disease, perceived to be at greater risk for adverse effects of short acting bronchodilators. Antibiotics and systemic steroids were given to 70% of the patients, and while steroids therapy continued at discharge (70.5%), antibiotics prescription dropped to 10%. This might be the result of the short 3-day course of azithromycin and the possible tendency to stop antibiotics once the patient improves, especially if there are no findings to support an infectious cause for the exacerbation. During AECOPD, chest radiography can contribute to the differential diagnosis and affect the management in up to 23% of the cases (3, 19). Spirometry, on the other hand, is not indicated during an exacerbation (19). In our study, 85% had a chest X-ray and 5% performed spirometry during admission, similar to rates presented in previous studies (17, 18).

Evidence regarding the role of LABD or ICS during an AECOPD is lacking. The 2022 GOLD guidelines recommend continuing these treatments during the exacerbation or start/re-initiate them as soon as possible before or upon hospital discharge (3). Lindenauer at el. described the inpatient use of LABD among 77,378 AECOPD patients and found much higher rates of LABD (41%) and ICS (40.6%) treatment compared with ours (20). Our results also show that LABDs were stopped during hospitalization for many patients, while the use of ICS significantly increased. Explanations for these observations are the use of nebulized ICS during hospitalization, the lack of LAMA and LABA inhalers in internal medicine departments and low awareness of the staff to the importance of continuation of background therapy. An important observation is the low rate of LABDs use prior to the admission (47%), which highlights another important gap that should be addressed at the ambulatory setting. In addition, the association of LABD with lower 1-year mortality is in line with the studies above, although causality could not be imply based on the design of our study.

The GOLD guidelines recommend initiating treatment based on ABCD assessment tool (3). Using this tool, 78% of our patient population should be labeled in groups C or D prior their hospitalization, and 100% after their discharge. In GOLD C and D groups, LABD is the recommended treatment. The effect of these drugs should not be underestimated. LABAs and LAMAs significantly reduce exacerbations rate and improve lung function and health status (21). Their concurrent use is superior to either alone (22), and studies from recent years demonstrated an even higher benefit from triple therapy with an ICS (23, 24), leading to reduced mortality in this specific population. Our survey found that LABDs were prescribed to only 54% of the patients upon discharge. Yip et al. showed an even lower prescription rate of 42.5% (17). Furthermore, the low prescription rates of ICS/LABA (40.2%) should be highlighted, as it can also negatively affect patients’ frequency of exacerbations, admissions and mortality (23, 24).

Although LAMA therapy results in reduced exacerbations compared with LABA (25), it was substantially underused in our survey, prescribed to only 27.5% at discharge. One of the main reasons may be that until 2020, a pulmonologist recommendation was required for the reimbursement of LAMA in Israel. However, the rate of combined ICS/LABA inhaler prescriptions was also low (42%), although no reimbursement issues present for such treatment, suggesting low awareness of the treating physicians regarding the benefit of guideline-recommended pharmacological therapy.

We found major gaps in the non-pharmacological recommendations as well. Pulmonary rehabilitation was shown to be the most effective intervention to improve symptoms and may also reduce hospitalizations among patients with previous exacerbations (3, 26, 27). In our cohort, a referral for pulmonary rehabilitation program was prescribed to only 11% of the patients, possibly due to a lack of awareness to its availability in Israel and importance. The GOLD guidelines state that physicians should emphasize the importance of a smoke-free environment in every medical encounter (3). Strategies for smoking cessation have a success rate of up to 25% and impact the natural history of the disease (28). In addition, counseling by physicians significantly increases quit rates compared to self-initiated strategies (29). Smoking cessation was recommended to only 43% of active smokers in our survey, and referral to smoking cessation program was recommended to only 13.5%. These low rates might again reflect a lack in awareness of the impact of such interventions.

COPD exacerbations cause a major burden on the patient and healthcare system (30). Measures are needed to address the gaps we describe for adequate treatment and prevention of future events. One possible measure is to define the different aspects of inpatient care of AECOPD as quality indicators (31). Doing so will create an incentive for improvement in this area with more resources invested. Moreover, quality indicators in the ambulatory settings prior to AECOPD hospitalization, may improve patient adherence and outcomes. Pre-discharge care bundles, that showed mixed results among AECOPD patients, were proven effective for a variety of other conditions like ventilator associated pneumonia and heart failure, and by being more specified could lead to better outcomes (32, 33). Other interventions such as early involvement of a respiratory specialist and the routine participation of respiratory coordinators may improve inpatient care and the utilization of the recommended post hospitalization interventions.

The strengths of our survey are that it likely represents the inpatient care of AECOPD in Israel, given the high numbers of included patients, departments, and medical centers. Moreover, our survey characterizes the outpatient COPD management in Israel prior to the exacerbation leading to hospitalization. Our findings highlight the current gaps in the care of AECOPD, not only in the acute setting but also in the ambulatory setting prior admission and at discharge.

The limitations include the fact that most of the data was retrospective and that prior spirometry test results could not be assessed to confirm the diagnosis of COPD and determine the severity of airflow limitation, as they were not captured in the COPDIS database. However, as the main condition of all patients was considered to be AECOPD by their treating physicians, the above limitation should not influence the gaps encountered in our study. Furthermore, enrolling patients to the prospective cohort was at the discretion of the medical teams and according to their ability to sign the informed consent and provide clinical data, which means that a selection bias could not be ruled out. We tried to minimize such bias by the wide distribution of the study in different departments and medical centers nationally, in a variety of communities.

In conclusion, this study provides an overview of the characteristics and care of patients with AECOPD that were admitted to an internal medicine department in Israel 2017–2019. We found gaps in the ambulatory, in-hospital, and pre-discharge treatment, that should be addressed. We identified significant challenges regarding the discharge plan and continuation of care after this meaningful and potentially fatal event. Further research is needed to assess the impact different interventions, before, during and after an AECOPD, on clinical practice and patient outcomes.

## Data availability statement

The datasets presented in this article are not readily available because request for the dataset supporting our results can be made by to the corresponding author and will be given after approval of the institutes ethics committee. Requests to access the datasets should be directed to Ophir068@gmail.com.

## Ethics statement

The studies involving humans were approved by the institutional review board of each included center (Sourasky medical center, Hadassah medical center, Carmel medical center, Galilee medical center, Rabin medical center, Rambam medical center, Soroka medical center, Poriya medical center, Shaare Zedek medical center, Sheba medical center, Shamir medical center, Barzilai medical center). The studies were conducted in accordance with the local legislation and institutional requirements. The participants in the prospective cohort provided their written informed consent to participate in this study. Informed consent was waved for the retrospective cohort given its design by the institutional review board.

## Author contributions

AB-S is the co-chair of the COPDIS steering committee, designed the study and wrote the manuscript. OF wrote the manuscript and designed the figures. TO and RK performed data acquisition and data analysis. MS is a member of the COPDIS steering committee, contributed to the study design and data analysis. AE designed the study and is the co-chair of the COPDIS steering committee. All authors were responsible for revising and approving the final submitted manuscript.

## Funding

This study received funding from the Israeli Ministry of Health, GlaxoSmithKline, Boehringer Ingelheim, AstraZeneca, Novartis, Kamada and Pfizer. The funders were not involved in the study design, collection, analysis, interpretation of data, the writing of this article or the decision to submit it for publication. All authors declare no other competing interests.

## Conflict of interest

The authors declare that the research was conducted in the absence of any commercial or financial relationships that could be construed as a potential conflict of interest.

## Publisher’s note

All claims expressed in this article are solely those of the authors and do not necessarily represent those of their affiliated organizations, or those of the publisher, the editors and the reviewers. Any product that may be evaluated in this article, or claim that may be made by its manufacturer, is not guaranteed or endorsed by the publisher.
